# Supplementary material for: A Theoretical Framework for Self-Supervised MR Image Reconstruction Using Sub-Sampling via Variable Density Noisier2Noise
Source: IEEE Trans Comput Imaging. Author manuscript; Available in PMC 2023 Aug 18. (PMC7614963; doi:10.1109/TCI.2023.3299212)
Supplement: Supplementary Material [file EMS184946-supplement-Supplementary_Material.pdf]

Supplementary material for "A theoretical framework for self-supervised MR image reconstruction using sub-sampling via variable density Noisier2Noise"

Charles Millard and Mark Chiew

Results on 1D sampled brain data at $R = 4$

Figures S1 and S2 show the methods' performance on the test set when R_Λ is tuned. The medians are shown in Table S1. Like figures 3 and 4 of the main paper, the distribution closest to fully supervised is K-weighted 1D partitioned SSDU with y_s input.

Fig. S3 shows a reconstruction example from the test set. Although 1D partitioned SSDU outperforms 2D partitioned SSDU quantitatively, both offer high quality reconstructions with minimal reconstruction artifacts. This contrasts with Fig. 4 of the main paper, where there is a clear qualitative difference between the reconstruction quality. This indicates that the benefit of using the same type of distribution of M_Λ as M_Ω is less substantial for less ambitious acceleration factors. Further, K-weighting SSDU only marginally improves the method's performance qualitatively. This is due to $(\mathbb{1} - K)^{-1}$ have smaller entries when $R_\Omega = 4$, so is a less crucial sampling compensation.

Fig. S4 shows the dependence of the self-supervised methods on R_Λ at $R_\Omega = 4$. As in the $R_\Omega = 8$ plot in Fig. 6 of the main paper, K-weighted 1D partitioned SSDU is the most robust, and 2D partitioned SSDU is very sensitive to the tuning of R_Λ, especially for the estimate with y_s input.

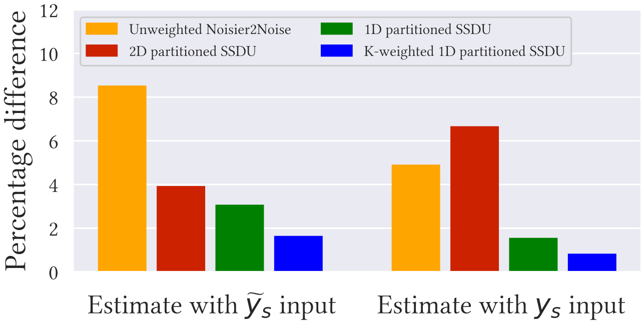

Figure S1: The percentage difference between fully supervised method and the mean NMSE for all methods at $R_\Omega = 4$. Here, M_Ω is 1D distributed and R_Λ has been tuned to minimize the test set NMSE.

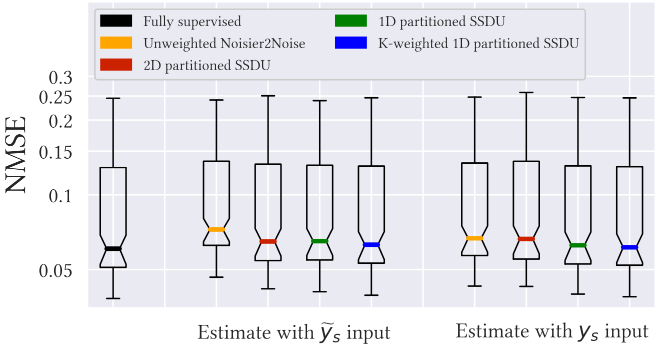

Figure S2: The NMSE and SSIM for all methods at $R_\Omega = 4$, where R_Λ has been tuned to minimize the test set NMSE. 1D partitioned SSDU was found to perform the most similarly to fully supervised training. The exact numerical values are in Table S1.

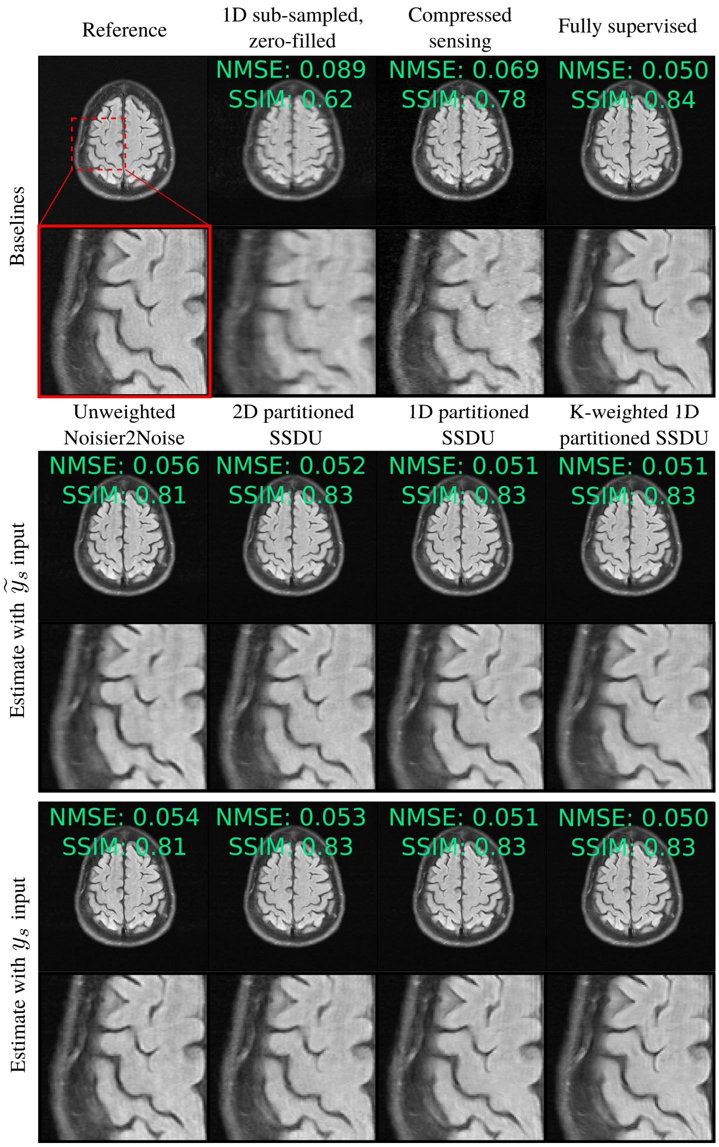

Figure S3: A reconstruction example at $R_\Omega = 4$, with a tuned R_Λ. As in Fig. 5 of the main paper, K-weighted 1D partitioned SSDU has the best score. However, there is a less substantial difference between the estimates.

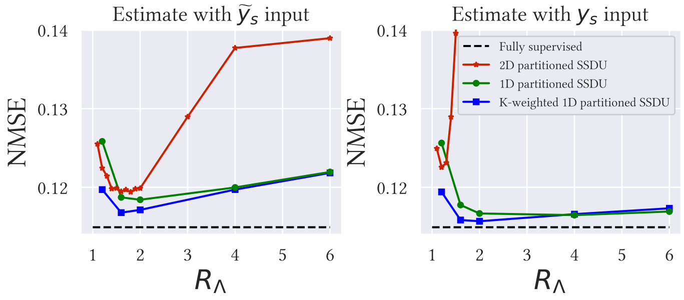

Figure S4: The dependence of the test set NMSE on R_Λ at $R_\Omega = 4$ for both outputs.

SUB-SAMPLING FACTOR R_Ω	4		8	
NETWORK INPUT AT INFERENCE	$\widetilde{y}_s$	y_s	$\widetilde{y}_s$	y_s
Fully supervised	-	0.061	-	0.077
Unweighted Noisier2Noise	0.073	0.067	0.096	0.097
2D partitioned SSDU	0.065	0.066	0.083	0.089
1D partitioned SSDU	0.067	0.063	0.084	**0.078**
K-weighted 1D partitioned SSDU	0.063	**0.061**	0.082	**0.078**

Table S1: The median NMSE on the test set for all methods, where R_Λ is tuned. The best result from the self-supervised methods is highlighted in bold.

Table of median NMSE results on the test set

For reference, Table S1 shows the median NMSE scores on the test set for tuned R_Λ. In other words, it shows the numerical values of the colored horizontal lines in Fig. 4 of the main manuscript and Fig. S2 for $R_\Omega = 8, 4$ respectively.
