# Supplementary material for: A Theoretical Framework for Self-Supervised MR Image Reconstruction Using Sub-Sampling via Variable Density Noisier2Noise
Source: IEEE Trans Comput Imaging. Author manuscript; Available in PMC 2023 Aug 18. (PMC7614963; doi:10.1109/TCI.2023.3299212)
Supplement: Appendix [file EMS184946-supplement-Appendix.pdf]

Appendix

A. Proof of Variable Density Noisier2Noise

This section of the Appendix proves that when $p_j \neq 0$ and $\widetilde{p}_j \neq 1$ for all j,

$$\mathbb{E}[Y_0|\widetilde{Y}] = (\mathbb{1} - K)^{-1}(\mathbb{E}[Y|\widetilde{Y}] - K\widetilde{Y}), \tag{18}$$

where $K = (\mathbb{1} - \widetilde{P}P)^{-1}(\mathbb{1} - P)$ for $P = \mathbb{E}[M_\Omega]$ and $\widetilde{P} = \mathbb{E}[M_\Lambda]$.

Proof: This proof is based on Section III-D of Noisier2Noise [41], but with more mathematical detail and generalized to variable density sampling. Following the compressed sensing literature, this article uses p_j to refer to the probability that the jth location in k-space is sampled. This differs to [41], which uses p to denote the probability that a pixel is *zeroed*.

We wish to compute $\mathbb{E}[Y_j|\widetilde{Y}_j]$ as a function of $\mathbb{E}[Y_{0,j}|\widetilde{Y}_j]$. To do this, we split $\mathbb{E}[Y_j|\widetilde{Y}_j]$ into two cases, for conditions $\widetilde{Y}_j \neq 0$ or $\widetilde{Y}_j = 0$, and subsequently construct an expression that is consistent with both.

Case 1. $(\mathbb{E}[Y_j|\widetilde{Y}_j \neq 0])$*:* By the measurement model $\widetilde{Y} = M_\Lambda Y = M_\Lambda M_\Omega Y_0$, the singly sub-sampled Y_j must take the same value as $\widetilde{Y}_j$ when $\widetilde{Y}_j \neq 0$. Therefore

$$\mathbb{E}[Y_j|\widetilde{Y}_j \neq 0] = \widetilde{Y}_j. \tag{19}$$

Case 2. $(\mathbb{E}[Y_j|\widetilde{Y}_j = 0])$*:* Using the partition theorem for expectations, we write $\mathbb{E}[Y_j|\widetilde{Y}_j = 0]$ as the weighted sum of $\mathbb{E}[Y_j|\widetilde{Y}_j = 0 \cap Y_j = 0]$ and $\mathbb{E}[Y_j|\widetilde{Y}_j = 0 \cap Y_j \neq 0]$:

$$\begin{aligned}\mathbb{E}[Y_j|\widetilde{Y}_j = 0] = \mathbb{E}[Y_j|\widetilde{Y}_j = 0 \cap Y_j = 0] \cdot k_j \\ + \mathbb{E}[Y_j|\widetilde{Y}_j = 0 \cap Y_j \neq 0] \cdot (1 - k_j),\end{aligned} \tag{20}$$

where we define $k_j = \mathbb{P}[Y_j = 0|\widetilde{Y}_j = 0]$. Evaluating each of the terms on the right-hand-side of (20) in turn:

- $\mathbb{E}[Y_j|\widetilde{Y}_j = 0 \cap Y_j = 0]$: Since the random variable Y_j is conditionally zero, its expectation is also zero:

$$\mathbb{E}[Y_j|\widetilde{Y}_j = 0 \cap Y_j = 0] = 0.$$

- $\mathbb{E}[Y_j|\widetilde{Y}_j = 0 \cap Y_j \neq 0]$: The measurement model implies that when Y_j is non-zero, and therefore unmasked, it takes the value of $Y_{0,j}$. Therefore its expectation can be written in terms of the expectation of $Y_{0,j}$:

$$\mathbb{E}[Y_j|\widetilde{Y}_j = 0 \cap Y_j \neq 0] = \mathbb{E}[Y_{0,j}|\widetilde{Y}_j = 0]. \tag{21}$$

- k_j: By the definition of conditional expectation:

$$k_j = \mathbb{P}[Y_j = 0|\widetilde{Y}_j = 0] = \frac{\mathbb{P}[Y_j = 0 \cap \widetilde{Y}_j = 0]}{\mathbb{P}[\widetilde{Y}_j = 0]}.$$

The numerator is

$$\begin{aligned}\mathbb{P}[Y_j = 0 \cap \widetilde{Y}_j = 0] &= \mathbb{P}[Y_j = 0] \\ &= 1 - p_j,\end{aligned}$$

where $p_j = \mathbb{P}[Y_j \neq 0] = \mathbb{E}[M_{\Omega,jj}]$ is the probability that $j \in \Omega$. By the partition theorem, the denominator is

$$\begin{aligned}\mathbb{P}[\widetilde{Y}_j = 0] &= \mathbb{P}[\widetilde{Y}_j = 0|Y_j = 0]\mathbb{P}[Y_j = 0] \\ &\quad + \mathbb{P}[\widetilde{Y}_j = 0|Y_j \neq 0]\mathbb{P}[Y_j \neq 0] \\ &= 1 \cdot (1 - p_j) + (1 - \widetilde{p}_j)p_j \\ &= 1 - \widetilde{p}_j p_j,\end{aligned}$$

where $\widetilde{p}_j = \mathbb{P}[\widetilde{Y} \neq 0] = \mathbb{E}[M_{\Lambda,jj}]$. Therefore

$$k_j = \mathbb{P}[Y_j = 0|\widetilde{Y}_j = 0] = \frac{1 - p_j}{1 - \widetilde{p}_j p_j}. \tag{22}$$

Substituting the above results into (20) gives

$$\mathbb{E}[Y_j|\widetilde{Y}_j = 0] = \mathbb{E}[Y_{0,j}|\widetilde{Y}_j = 0](1 - k_j), \tag{23}$$

where k_j is defined in (22).

Combining Cases 1 and 2. ($\mathbb{E}[Y_j|\widetilde{Y}_j]$)*:* To find $\mathbb{E}[Y_j|\widetilde{Y}_j]$, one must construct an expression that holds for both (19) and (23). Consider the following candidate:

$$\mathbb{E}[Y_j|\widetilde{Y}_j] = (1-k_j)\mathbb{E}[Y_{0,j}|\widetilde{Y}_j] + k_j\widetilde{Y}_j. \tag{24}$$

This expression can be verified as consistent with (19) by setting $\widetilde{Y}_j \neq 0$:

$$\begin{aligned}\mathbb{E}[Y_j|\widetilde{Y}_j \neq 0] &= (1-k_j)\mathbb{E}[Y_{0,j}|\widetilde{Y}_j \neq 0] + k_j\widetilde{Y}_j\\ &= (1-k_j)\widetilde{Y}_j + k_j\widetilde{Y}_j\\ &= \widetilde{Y}_j,\end{aligned}$$

as required. Secondly, setting $\widetilde{Y}_j = 0$ gives

$$\begin{aligned}\mathbb{E}[Y_j|\widetilde{Y}_j = 0] &= (1-k_j)\mathbb{E}[Y_{0,j}|\widetilde{Y}_j = 0] + k_j\cdot 0\\ &= (1-k_j)\mathbb{E}[Y_{0,j}|\widetilde{Y}_j = 0],\end{aligned}$$

as required by (23). Therefore (24) is consistent with both (19) and (23), so is a correct expression for $\mathbb{E}[Y_j|\widetilde{Y}_j]$.

When $1-k_j \neq 0$ we can rearrange (24) for $\mathbb{E}[Y_{0,j}|\widetilde{Y}_j]$:

$$\mathbb{E}[Y_{0,j}|\widetilde{Y}_j] = (1-k_j)^{-1}(\mathbb{E}[Y_j|\widetilde{Y}_j] - k_j\widetilde{Y}_j). \tag{25}$$

By the expression for k_j given in (22), $1-k_j$ is

$$1-k_j = 1 - \frac{1-p_j}{1-\widetilde{p}_j p_j} = \frac{p_j(1-\widetilde{p}_j)}{1-\widetilde{p}_j p_j},$$

so is non-zero when $p_j \neq 0$ and $\widetilde{p}_j \neq 1$. Writing (25) in terms of vectors and matrices yields (18), as required.

B. Proof of SSDU

This section of the Appendix proves that a network trained with SSDU's loss weighting $(\mathbb{1} - M_\Lambda)M_\Omega$ satisfies

$$(\mathbb{1} - K)(\mathbb{1} - M_\Lambda M_\Omega)(f_{\theta^*}(\widetilde{Y}) - \mathbb{E}[Y_0|\widetilde{Y}]) = 0. \tag{26}$$

Proof: By (6), the minimum of SSDU's loss function (12) gives a function that satisfies

$$\mathbb{E}[(\mathbb{1} - M_\Lambda)M_\Omega(f_{\theta^*}(\widetilde{Y}) - Y)|\widetilde{Y}] = 0 \tag{27}$$

Similarly to Section A of the Appendix, the following derives expressions for $\mathbb{E}[(\mathbb{1} - M_\Lambda)M_\Omega(f_{\theta^*}(\widetilde{Y}) - Y)|\widetilde{Y}]$ under two conditions, $\widetilde{Y}_j \neq 0$ and $\widetilde{Y}_j = 0$, and subsequently find an expression that is true for both. In the following, $\widetilde{m}_j$ and m_j are the jth diagonals of M_Λ and M_Ω respectively.

Case 1. ($\mathbb{E}[(1-\widetilde{m}_j)m_j(f_{\theta^*}(\widetilde{Y})_j - Y_j)|\widetilde{Y}_j \neq 0]$)*:* When $\widetilde{Y}_j \neq 0$, the jth entry is not masked: $\widetilde{m}_j = 1$. Therefore $(1-\widetilde{m}_j)m_j = 0$ and the expression is zero:

$$\mathbb{E}[(1-\widetilde{m}_j)m_j(f_{\theta^*}(\widetilde{Y})_j - Y_j)|\widetilde{Y}_j \neq 0] = 0. \tag{28}$$

Case 2. ($\mathbb{E}[(1-\widetilde{m}_j)m_j(f_{\theta^*}(\widetilde{Y})_j - Y_j)|\widetilde{Y}_j = 0]$)*:* When $\widetilde{Y}_j = 0$, $\widetilde{m}_j m_j = 0$, so $(1-\widetilde{m}_j)m_j = m_j$. Therefore

$$\begin{aligned}&\mathbb{E}[(1-\widetilde{m}_j)m_j(f_{\theta^*}(\widetilde{Y})_j - Y_j)|\widetilde{Y}_j = 0]\\ &\quad= \mathbb{E}[m_j(f_{\theta^*}(\widetilde{Y})_j - Y_j)|\widetilde{Y}_j = 0].\end{aligned} \tag{29}$$

As for Case 2 of Section A of the Appendix, we can use the partition theorem to express (29) as a weighted sum:

$$\begin{aligned}&\mathbb{E}[m_j(f_{\theta^*}(\widetilde{Y})_j - Y_j)|\widetilde{Y}_j = 0]\\ &\quad= \mathbb{E}[m_j(f_{\theta^*}(\widetilde{Y})_j - Y_j)|\widetilde{Y}_j = 0 \cap Y_j = 0]\cdot k_j\\ &\qquad+ \mathbb{E}[m_j(f_{\theta^*}(\widetilde{Y})_j - Y_j)|\widetilde{Y}_j = 0 \cap Y_j \neq 0]\cdot(1-k_j),\end{aligned} \tag{30}$$

where $k_j = \mathbb{P}[Y_j = 0|\widetilde{Y}_j = 0]$ as in Section A of the Appendix, given in (22). Taking each term in turn:

- $\mathbb{E}[m_j(f_{\theta^*}(\widetilde{Y})_j - Y_j)|\widetilde{Y}_j = 0 \cap Y_j = 0]$: Since $Y_j = 0$ when it is zeroed by the mask, $m_j = 0$. Therefore
$$\mathbb{E}[m_j(f_{\theta^*}(\widetilde{Y})_j - Y_j)|\widetilde{Y}_j = 0 \cap Y_j = 0] = 0.$$
- $\mathbb{E}[m_j(f_{\theta^*}(\widetilde{Y})_j - Y_j)|\widetilde{Y}_j = 0 \cap Y_j \neq 0]$: When $Y_j \neq 0$, it is not zeroed by the mask, so $m_j = 1$:
$$\begin{aligned}&\mathbb{E}[m_j(f_{\theta^*}(\widetilde{Y})_j - Y_j)|\widetilde{Y}_j = 0 \cap Y_j \neq 0]\\ &\quad= \mathbb{E}[f_{\theta^*}(\widetilde{Y})_j - Y_j|\widetilde{Y}_j = 0 \cap Y_j \neq 0].\end{aligned}$$
Further, since $Y_j = Y_{0,j}$ when $Y_j \neq 0$ by the measurement model,
$$\begin{aligned}&\mathbb{E}[f_{\theta^*}(\widetilde{Y})_j - Y_j|\widetilde{Y}_j = 0 \cap Y_j \neq 0]\\ &\quad= \mathbb{E}[f_{\theta^*}(\widetilde{Y})_j - Y_{0,j}|\widetilde{Y}_j = 0].\end{aligned}$$

Substituting the above results in to (30) gives

$$\begin{aligned}&\mathbb{E}[(1-\widetilde{m}_j)m_j(f_{\theta^*}(\widetilde{Y})_j - Y_j)|\widetilde{Y}_j = 0]\\ &\quad= \mathbb{E}[f_{\theta^*}(\widetilde{Y})_j - Y_{0,j}|\widetilde{Y}_j = 0]\cdot(1-k_j).\end{aligned} \tag{31}$$

Combining Cases 1 and 2: A correct expression for $\mathbb{E}[(1-\widetilde{m}_j)m_j(f_{\theta^*}(\widetilde{Y})_j - Y_j)|\widetilde{Y}_j = 0]$ must be true for both Case 1 and 2, so consistent with both (28) and (31). Consider the candidate

$$\begin{aligned}&\mathbb{E}[(1-\widetilde{m}_j)m_j(f_{\theta^*}(\widetilde{Y})_j - Y_{0,j})|\widetilde{Y}_j]\\ &\quad= (1-k_j)(1-\widetilde{m}_j m_j)\mathbb{E}[f_{\theta^*}(\widetilde{Y})_j - Y_{0,j}|\widetilde{Y}_j].\end{aligned} \tag{32}$$

Equation (32) is consistent with (28) because $(1-\widetilde{m}_j m_j) = 0$ when $\widetilde{Y}_j \neq 0$, and consistent with (31) because $(1-\widetilde{m}_j m_j) = 1$ when $\widetilde{Y}_j = 0$. Using the vector form of (32) and setting $\mathbb{E}[f_{\theta^*}(\widetilde{Y})|\widetilde{Y}] = f_{\theta^*}(\widetilde{Y})$ gives

$$\begin{aligned}&\mathbb{E}[(\mathbb{1} - M_\Lambda)M_\Omega(f_{\theta^*}(\widetilde{Y}) - Y)|\widetilde{Y}]\\ &\quad= (\mathbb{1} - K)(\mathbb{1} - M_\Lambda M_\Omega)(f_{\theta^*}(\widetilde{Y}) - \mathbb{E}[Y_0|\widetilde{Y}]) = 0,\end{aligned} \tag{33}$$

as required.
